# Time-effect relationship between spare-time exercise and sleep quality in middle school student by parallel latent growth and cross-lagged panel model

**DOI:** 10.3389/fpsyg.2025.1590573

**Published:** 2025-06-04

**Authors:** Ziming Wei, Mo Sha, Zixia Bu, Yujie Liu, Yue Gao, Fuqiang Dong, Shan Jiang, Da Huang

**Affiliations:** ^1^College of Physical Education and Sports, Beijing Normal University, Beijing, China; ^2^Department of Physical Education, Communication University of China, Beijing, China; ^3^School of International Chinese Language Education, Beijing Normal University, Beijing, China; ^4^Division of Sports Science and Physical Education, Tsinghua University, Beijing, China; ^5^School of Leisure Sports and Tourism, Beijing Sport University, Beijing, China; ^6^College of Physical Education, Minzu University of China, Beijing, China; ^7^Department of Sports Science and Physical Education, The Chinese University of Hong Kong, Shatin, China

**Keywords:** exercise, sleep quality, middle school student, parallel latent growth modeling, cross-lagged panel model

## Abstract

**Introduction:**

Sleep quality (SQ) is an important factor affecting the life and academic performance of secondary school students, and it has been found that spare-time exercise (STE) can improve SQ, but the psychological mechanism and timeliness have not been elucidated. Therefore, this study analyzed it using cross-lagged panel model (CLPM) and parallel latent growth modeling (PLGM).

**Methods:**

A total of 894 students from six secondary schools in Beijing, China, participated in the study. Participants were monitored over time through three 4-month intervals (T1, T2, T3) using the International Physical Activity Questionnaire (IPAQ) and the Pittsburgh Sleep Quality Index (PSQI). Data analysis was conducted using Pearson's test, CLPM, and PLGM.

**Results:**

Correlation results showed a significant negative correlation between STE and SQ across the 3 measures (*r* = [−0.31, −0.14]; *P* < 0.01). PSQI results showed a linear decreasing trend in STE (slope = −0.04, *P* < 0.01) and a linear increasing trend in SQ (slope = 0.02, *P* < 0.01) among secondary school students across the 3 time periods. CLPM results showed that the initial level of STE negatively predicted the initial level and subsequent growth rate of SQ (β = −0.20, *P* < 0.01). The decreasing rate of STE significantly predicted the later SQ growth rate β = −0.06, *P* < 0.01). (4) STE was a negative predictor of SQ (β = −0.17, *P* < 0.01).

**Conclusion:**

(1) Initial levels of STE in secondary school students negatively predicted the rate of development of SQ over the course of the study, and higher levels of STE may have been a protective factor in the development of SQ levels. (2) The effect of STE on SQ was more stable, negatively predicting SQ at the next time point for all three measurements. SQ, on the other hand, had no significant effect on changes in STE. (3) STE decreases over time, so continued exercise and intensity are key to improving SQ.

## Introduction

Sleep quality (SQ) has been identified as a primary issue affecting the mental health of contemporary secondary school students and a particular symptom of premature anxiety manifested by social adaptation (Scott et al., 2021). SQ is a gate for the difficulty of falling asleep and staying asleep at night, and the number of times awake during sleep. It is a combination of objective evaluations, such as the time to fall asleep and the number of times awake, and subjective evaluations, such as the difficulty of falling asleep and the degree of rest (Cheng et al., 2018). A plethora of studies have previously corroborated the notion that sleep disorders in secondary school students are closely related to a number of factors, including but not limited to excessive academic stress, interpersonal disorders, attention deficit and poor mood (Lanfredi et al., 2019; DuPaul et al., 2021). The concept of SQ for secondary school students encompasses the domains of long-term mental health and emotional stability. Poor SQ has been demonstrated to be a contributing factor to the development of various serious problems. According to surveys conducted by the World Health Organization (WHO), it is estimated that 27% of the global population experiences sleep difficulties, indicating that one in three individuals encounter challenges in achieving adequate sleep (Gao et al., 2024). The sleep situation of the Chinese population is a matter of concern. According to the 2024 report, the national average sleep time is 6.5 h, and 38.2 per cent of the population, or approximately 510 million people, are suffering from sleep problems that have a detrimental effect on quality of life and work efficiency, and may even lead to health hazards (Fang et al., 2024). For secondary school students, SQ concerns an individual's mental state and cognitive functioning, which directly affects their academic performance (Lewin et al., 2017). Consequently, enhancing the efficacy of SQ in secondary school students operating within constrained environments has emerged as a pivotal concern in the fields of psychology and neuroscience.

The WHO's Mental Health Gap Action Programme (mhGAP), published in 2023, suggests that physical activity (PA) is a non-pharmacological intervention that can be effective in improving SQ and enhancing mental health (Keynejad et al., 2021). The daily school schedule for high school students is relatively fixed, particularly for the final years of junior and senior high school, which leaves limited free time for physical education and PA during the school year. Consequently, students rely on space-time exercise (STE) to satisfy their PA desires. It has been demonstrated that STE is an extension of the school physical education curriculum, and that students utilize their leisure time outside of school for the purposes of health, recreation and entertainment. Concurrently, STE functions not only as a conduit for socialization among secondary school students, but also as a means of intervening in the SQ, enhancing mood, and improving quality of life (Takemura et al., 2024). The Neuroplasticity Theory, proposed by experts in the field of neuroscience (Lu et al., 2024), posits that long-term STE has the capacity to modify the plasticity of brain synapses, thereby accelerating the frequency and intensity of interactions between neurons and impacting the pathways of neuro The release of neurotransmitters such as dopamine and glutamate, along with the continuous stimulation of the hippocampus in the cerebral cortex and prefrontal lobes, among other regions, contributes to the establishment of a physiological cyclic pattern. This, in turn, has been shown to enhance the efficiency of sleep nerve conduction (De Miguel et al., 2021; Starling, 2022; Blume and Royes, 2024). For example, from a neurotransmitter perspective, STE may promote the onset and maintenance of Non-Rapid Eye Movement (NREM) sleep (Halson, 2014). From a brain structure perspective, STE increases the functional connectivity of the thalami-cortical pathway and prefrontal cortex to re-model the neural circuits associated with sleep-wake regulation and improve sleep depth and stability (Lee et al., 2025). In the intracranial environment, STE can accelerate cerebrospinal fluid flow, activate the glymphatic system, increase the efficiency of removing metabolic waste such as amyloid beta protein, and optimize the neuronal microenvironment and sleep quality (Lee et al., 2025).

A plethora of studies have demonstrated that secondary school students' participation in STE is not only efficacious in enhancing their mental state and quality of life, but also improves SQ and negative emotions, and ultimately improves an individual's academic performance and cognitive functioning. Javelle's meta-analysis demonstrated that secondary school students' participation in STE for more than half an hour per day can improve their cognitive regulation of false emotions and develop positive outlooks on life and emotional values (Javelle et al., 2023). In addition, Wiklund's findings indicated that secondary school student populations lacking STE exhibited a range of behavioral and emotional tendencies, including withdrawn and apathetic tendencies, as well as impaired motivation. These students demonstrated an inadequate ability to regulate negative emotional responses and frequently experienced persistent sleep disorders. The overall impression was one of haggardness, depression, and anxiety (Wiklund et al., 2018). Furthermore, a subsequent survey study by Jordan discovered that external environmental discomfort is a significant factor impacting secondary school students' emotional expression, in addition to its role in the development of sleep disorders. The study also demonstrated that developmental changes in STE can be sustained and effectively influence subsequent SQ issues (Jordan et al., 2023). Therefore, it can be hypothesized that middle school student STE is likely to have a negative predictive effect on STE.

The present study was predicated on extant literature, which posits the hypothesis that STE plays an important role in the lives of secondary school students as a means of improving SQ and mental health. Nevertheless, there remains a paucity of longitudinal evidence to elucidate the time-spanning characteristics of STE in improving secondary school students' SQ. In light of the linear trends and significant levels of STE and SQ at the baseline level among secondary school students observed in previous studies, a reduction in time-spanning research was deemed necessary to prevent subject attrition and address systematic difficulties in the collection of data. The present study adopted a longitudinal tracking study spanning 1 year, which was conducted on three occasions. The study utilized an unconditional latent growth curve model, a parallel latent growth modeling (PLGM) approach, and a cross-lagged panel model (CLPM). The objective of these analyses was to investigate the developmental trajectories and causal relationships between STE and SQ in secondary school students. In light of the aforementioned findings, the following hypotheses were put forward in this study: (I) Secondary school students' STE levels are prone to a linear decline over time. (II) The initial level of secondary school students' STE has a negative correlation with the developmental changes of secondary school students' SQ. (III) Secondary school students' STE levels exhibit a negative correlation with secondary school students' SQ in subsequent time periods.

## Materials and methods

### Participants

A random whole-cluster sampling method was employed to select six secondary schools in Beijing for this study. The class teachers who accompanied the class for consultation and guidance were responsible for explaining the subject questionnaire to the school's secondary students, who were the object of investigation. The accompanying questionnaire was distributed in the physical education class of the participants, in accordance with pre-agreed tracking research multiple cycles. A snowballing approach was used, with class-based cluster sampling, to exclude individuals with physical movement disorders, emotional adjustment disorders, sleep disorders, and those who were unwilling to participate. The initial phase of experimentation (T1) commenced in early March 2024, the subsequent phase (T2) was conducted in July 2024, and the third phase (T3) was completed in November 2024. Participants were requested to identify themselves prior to and following the process. A total of 894 valid responses were obtained for the initial measurement, 779 for the second, and 651 for the third. Participant demographics are detailed in Table 1.

**Table 1:** Participants' physical characteristics.

**Parameter**		**Value**
Male		552
Female		342
Age (yr)		16.74 ± 2.37
BMI (kg/m2)		24.58 ± 1.45
Screen usage time (h/day)		4.89 ± 2.21
Sedentary behavior (h/day)		8.34 ± 1.13
MET (/day)		< 2.8
Sleeping time (h/day)		7.18 ± 1.06
History of exercise	Yes	65%
	No	35%
Only child family	Yes	79%
	No	21%

In light of the longitudinal approach to data analysis, it is imperative to undertake a statistical examination of the sample attrition rate. Specifically, the attrition rate was recorded as 12.86 per cent for the initial data set and 16.43 per cent for the subsequent data set. A comparative analysis of the attrition rates and the disparities in the various demographic variables indicated that the overall distribution of the data was not normal, but rather skewed. The analysis of differences in individual demographic variables was conducted by employing and citing the rank sum test to ascertain the variability of the data for the group of subjects who ultimately participated on all three occasions. The analysis revealed no significant differences in attrition rates among the three subjects based on gender, grade, or year (*p* > 0.05), indicating that attrition rates in the sample study aligned with the study's requirements.

### Procedures

In the present study, three longitudinal follow-up surveys were conducted between March 2024 and November 2024 for the entire sample, with the indicators including STE and SQ. During the testing process, all indicators were collected and distributed through SoJum Software (Tencent Holdings Ltd.). Prior to data collection, the staff members elucidated the purpose and methodology of the survey to the respondents in person, addressing any queries they might have had. The respondents completed the survey with the consent of their counselors (class teachers) and their own consent. The survey was completed anonymously, without the involvement of personal information such as identification numbers or student numbers, and excluding invalid responses, brief responses (less than 200 s), responses with the same 10 consecutive choices, and responses with a high degree of homogeneity.

### Measurements

#### Spare-time exercise evaluation

Spare-time exercise was measured using the short version of International Physical Activity Questionnaire (IPAQ) (Bassett, 2003). IPAQ assigned metabolic equivalent (MET) corresponding to different intensities of PA by asking the study subjects about the frequency of the week (d/wk) and the time of day (min/d) for that intensity. The MET was 3.3 for walking, 4.0 for MPA, and 8.0 for VPA. The PA level for each intensity and the total PA level were calculated separately according to the formula. The IPAQ has been widely used in previous studies to measure STE participation profiles and daily PA levels in different age groups and also has high reliability (0.70–0.90) (Lee et al., 2011; Craig et al., 2003). In the three tests of pre- and post-test reliability of the questionnaire under investigation, the entries measuring the PA rating scale were not subject to censorship on account of the subjects' requirement of three entries for each construct. Cronbach's alpha coefficients for the three pre- and post-tests of the scale ranged from 0.71 to 0.79, thus meeting the subjects' requirements.

#### Sleep quality assessment

The Pittsburgh Sleep Quality Index (PSQI) scale, developed by Buysse, was utilized to assess sleep quality (Lehner et al., 2022). The SQ of secondary school students was measured using a scale comprising 19 measurement entries across seven dimensions. The scores for each entry are assessed on a scale of 0–3 points, and the scale as a whole adopts the reverse scoring method. Higher overall scores indicate more severe SQ problems, with scores generally ranging from 0–21 points. The SQ questionnaire has been employed in numerous studies involving healthcare professionals, high-pressure groups, the elderly, and individuals with sleep disorders. The reliability levels of the questionnaire have been reported to range from 0.70 to 0.90, which is consistent with the criteria established for social science research (Vetter and Cubbin, 2019). In the present study, the Cronbach a coefficient for the three pre- and post-scales ranged from 0.73 to 0.85, indicating that the reliability of the subjects was up to the mark.

#### Cross-lagged panel model construction

The cross-lagged panel model (CLPM) will be constructed based on the results of the three measurements by taking three longitudinal follow-up measurements of all participants at the following time points: T1: March 2024, T2: July 2024, T3: November 2024. Specifically, the three measures of IPAQ will be used as the primary variable and SQ as the secondary variable to observe the positive and negative predictive effects of STE on SQ. According to Andersen's method, the consistency of the three measurements and results of STE and SQ will be tested. And, in the order of baseline equivalence, loadings equivalence and intercept equivalence, PLGM and CLPM were constructed for secondary school students, respectively (Andersen, 2022).

### Statistical analysis

The present study is chiefly dependent on software packages such as IBM SPSS Statistics_26 and AMOS 24.0 in order to analyses the data with regard to descriptive statistics, trend development trajectory, and causality dialectic. Initially, the descriptive demographic variable difference comparison of each variable is conducted, primarily utilizing the sample *t*-test, rank sum test, and analogous methods, with the objective of ascertaining the presence of any disparities in demographic variables. This serves as a foundational element for the subsequent study, thereby delineating the direction of the investigation. The rank sum test is employed to quantify the attrition rate in the context of demographic differences. Secondly, Spearman's correlation coefficient matrix analysis was utilized to ascertain the significant degree of correlation between SQ and STE in the three time periods: T1, T2, and T3. Thereafter, the PLGM of the two variables was established in order to observe the trend of the unconditional latent growth curve model and the trajectory trend of the two in the parallel growth process, as well as the causal predictive relationship. Finally, a cross-lagged panel test was conducted with three times data to further determine the causal relationship between STE and SQ. The estimation of model fit parameters was mainly based on the Maximum Likelihood Estimate method. The reference standard of model fit index was based on Hu and Bentler's suggestion to screen the systematic bias caused by the same methodological bias, and the Harman one-way test was conducted for T1, T2, and T3 respectively. The results confirmed that the variance of the 1st factor loading was less than 40%, which was within the range specified by the scholars, and indicated that no significant common methodological bias existed in this study.

## Results

### Common method bias test and correlation analysis

The Harman one-way test was used to test the data for the presence of common method bias 23. In order to test whether the data were subject to systematic bias due to common method bias, a total of three tests were conducted. One-way tests were conducted for the T1, T2, and T3 phases, respectively. The results confirmed that the variance of the loadings of the 1st factor tested each time was less than 40%, which is within the range specified by the scholars, indicating that there is no significant bias in this study.

The results of the correlation test showed that the mean, standard error, and correlation coefficient matrices of STE and secondary school students' SQ for the three measurements before and after are shown in Table 2. From T1 to T2 and T3, the STE level showed an overall downward trend, while the SQ of secondary school students showed an upward trend. Moreover, there was a significant negative correlation between STE and secondary school students' SQ for the three measures (*r* = [−0.31, −0.14]; *P* < 0.01).

**Table 2:** Correlation coefficient matrix between spare-time exercise and sleep quality.

**Variable**	**M ±SD**	**T1-spare-time exercise**	**T2-spare-time exercise**	**T3-spare-time exercise**	**T1-sleep quality**	**T2-sleep quality**	**T3-sleep quality**
T1-spare-time exercise	0.27 ± 0.22	1					
T2-spare-time exercise	0.19 ± 0.24	0.31^**^	1				
T3-spare-time exercise	0.21 ± 0.20	0.26^**^	0.29^**^	1			
T1-sleep quality	0.35 ± 0.33	−0.28^**^	−0.17^**^	−0.18^**^	1		
T2-sleep quality	0.38 ± 0.31	−0.14^**^	−0.23^**^	−0.19^**^	0.37^**^	1	
T3-sleep quality	0.33 ± 0.32	−0.16^**^	−0.16^**^	−0.31^**^	0.39^**^	0.36^**^	1

** *P* < 0.01.

All variables have been standardized.

### Characteristics of developmental trajectory of spare-time exercise on middle school students

Constructing an unconditional linear latent variable growth model for secondary school students' STE to test the trend of overall STE. The fit index: χ^2^/*df* = 2.87, *P* = 0.18, CFI = 0.93, TLI = 0.91, RMSEA = 0.07, SRMR = 0.03, the overall fit index of the model is up to the standard, and the model parameters are detailed in Table 2.

**Figure 1:**
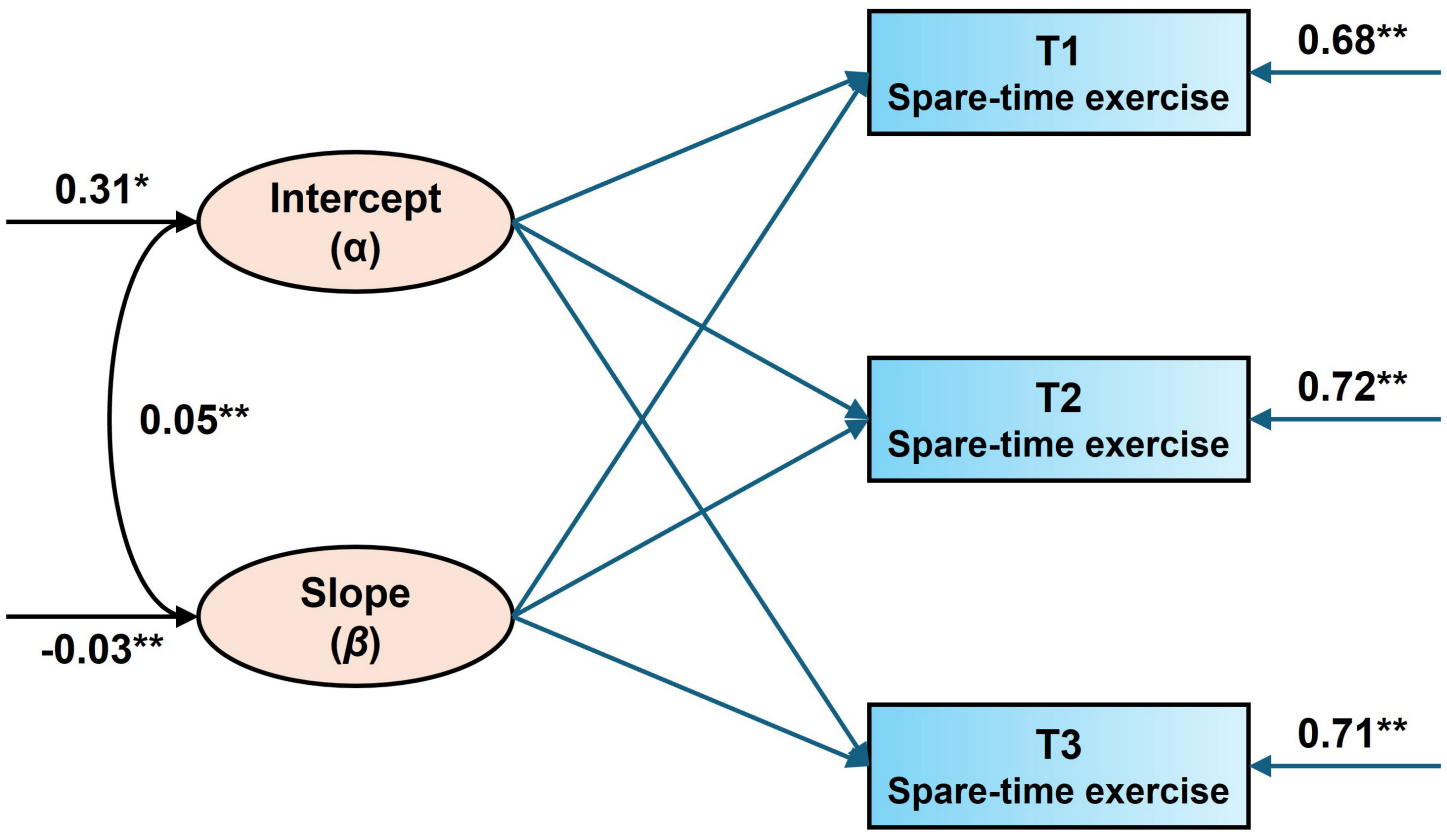
Changing trajectories of middle school students' spare-time exercise. All parameters are standardized path coefficients, * indicates *P* < 0.05, ** indicates *P* < 0.01.

The linear unconditional growth model showed an initial level of 0.31 (SE = 0.03, *P* < 0.001) for physical activity (standardized values) among secondary school students; there was a downward trend in STE participation among secondary school students (β = −0.04, SE = 0.01, *P* < 0.001), and the current results were consistent with research hypothesis (I). It was also found that the coefficients of variation for the intercept term (σ^2^ = 0.88, SE = 0.01, *P* < 0.001) and the slope (σ^2^ = 0.39, SE = 0.03, *P* < 0.001) were both significant at greater than 0. This suggests that there are individual and systematic variations in both the initial level of secondary school students' STE and their subsequent physical activity participation over time. Finally, the correlation coefficients of interception and slope were significant (*r* = −0.05, *P* < 0.001), i.e., the group with a high initial level of exercise among secondary school students, the faster the downward trend of participation in physical activity was in the subsequent three observations. Detailed information is shown in Figure 1.

### Characteristics of time-varying change in sleep quality on middle school students

This study attempted to construct an unconditional linear latent variable growth model of SQ for secondary school students to test the overall time period trend. The overall fit indices of the model were met as the fit indices χ^2^/*df* = 2.93, *P* = 0.07, CFI = 0.97, TLI = 0.98, RMSEA = 0.04, SRMR = 0.03. The details of the model parameters are shown in Table 3. The initial level of SQ (standardized value) for secondary school students in the linear unconditional growth model was 0.39 (SE = 0.04, *P* < 0.001). The SQ of secondary school students showed an increasing trend (β = 0.02, SE = 0.02, *P* < 0.001), and the current results were consistent with the research hypothesis (II).

**Table 3:** Parameter estimation for parallel latent growth modeling of spare-time exercise and sleep quality.

**Model**	**Coefficient**		**Variation**		**Slope-intercept**
	**Intercept**	**Slope**	**Intercept**	**Slope**	
Spare-time exercise	0.31^**^	−0.04^**^	0.88^**^	0.39^**^	−0.05^**^
Sleep quality	0.39^**^	0.02^**^	0.65^**^	0.03^**^	−0.02^**^

** *P* < 0.01.

**Figure 2:**
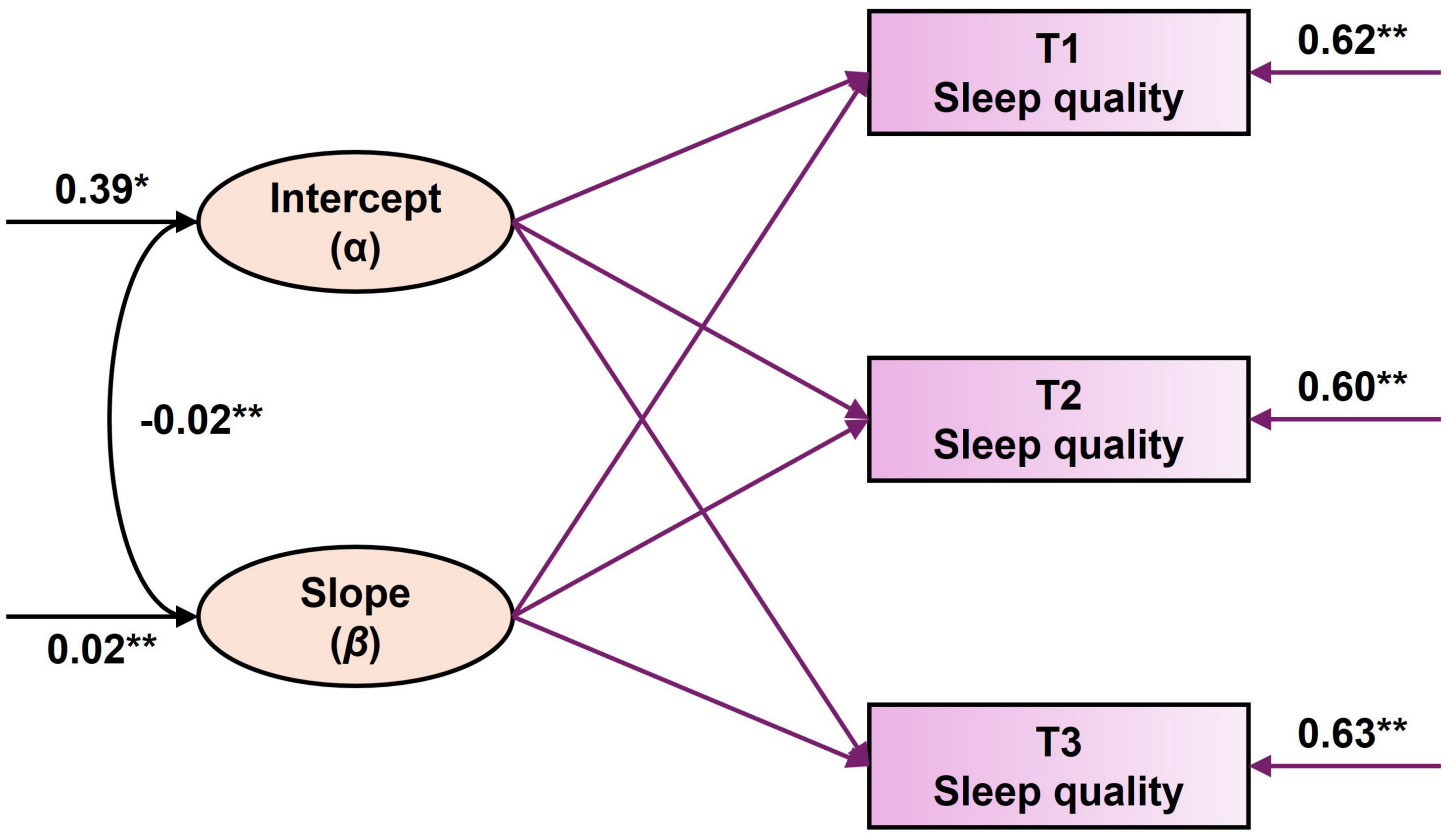
Changing trajectories of middle school students' sleep quality. All parameters are standardized path coefficients, * indicates *P* < 0.05, ** indicates *P* < 0.01.

It was also found that the coefficients of variation for the intercept term (σ^2^ = 0.65, SE = 0.03, *P* < 0.001) and the slope (σ^2^ = 0.03, SE = 0.04, *P* < 0.001) were both significantly greater than 0, suggesting that there were individual systematic differences in the initial level of secondary school students' SQ and in the subsequent changes in SQ over time. Finally, the correlation coefficients of intercept and slope were significant (*r* = −0.02, *P* < 0.001), i.e., a group with a high initial level of SQ among secondary school students had a slower rise in SQ over the three subsequent observations. Detailed information is shown in Figure 2.

### Parallel latent growth modeling of spare-time exercise and sleep quality on middle school students

**Figure 3:**
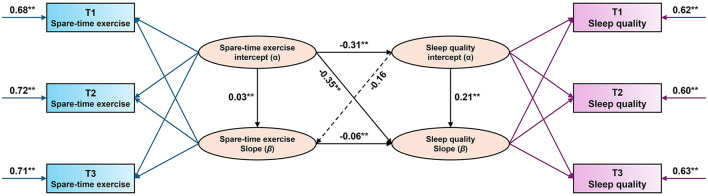
Parallel latent growth modeling of spare-time exercise and sleep quality in middle school students. All parameters are standardized path coefficients, ** indicates *P* < 0.01.

To further explore the association between STE and SQ, an attempt was made to construct a parallel growth model while simultaneously examining the overall profile of the slope and intercept terms of the two variables over time. The intercept term and slope of secondary school students' STE were used to predict the linear growth trend of SQ. The overall fit index of the model is good with χ^2^/*df* = 2.30, *P* = 0.07, CFI = 0.92, TLI = 0.99, RMSEA = 0.06, SRMR=0.04. Detailed information is shown in Figure 3.

In the latent variable parallel growth model of secondary school students' STE and SQ, the regression coefficient of the intercept of secondary school students' STE on the intercept of secondary school students' SQ was significant (β = −0.31, SE = 0.07, *P* < 0.001). This indicates that the lower the initial level of secondary school students' STE, the higher the initial level of secondary school students' SQ, and the results are consistent with the research hypothesis (III). It was also found that the intercept of secondary school students' STE negatively predicted the slope of secondary school students' SQ (β = −0.35, SE = 0.05, *P* < 0.001). This suggests that the lower the level of secondary school students' participation in STE, the faster secondary school students' SQ developed over subsequent time. It was also found that the slope of STE in secondary school students negatively predicted the slope of sleep disturbance in secondary school students (β = −0.06, SE = 0.05, *P* < 0.001). This suggests that with time fluctuations, the enthusiasm of secondary school students to participate in physical activity decayed the faster the SQ grew.

In order to further explore the causal association between STE and SQ of secondary school students and the magnitude of internal effects, and to avoid falling into the trap of preconceived theories, which may cause the real theoretical results to be deviated and lost. A parallel growth model of secondary school students' SQ and STE was established by attempting to swap dependent variables for the latent variable parallel growth model test. The results showed that the primary path of secondary school students' SQ predicting the STE model was not significant, and the regression coefficient of the intercept of secondary school students' SQ on the slope of STE was not significant (β = −0.16, SE = 0.22, *P* < 0.191). Therefore, it can be concluded that the middle school student SQ does not predict STE in the 3 before and after measurements.

### Construction of cross-lagged panel model based on three stages

The latent variable model can further observe the dynamic trajectory of the variables. In order to further clarify the sequential causal relationship between secondary school students' STE and SQ over time and to broaden the intrinsic mechanism between the two variables, based on Martens and Haase's suggestion, three times of longitudinal data were selected to conduct cross-lagged regression analyses of secondary school students' STE and SQ.

**Figure 4:**
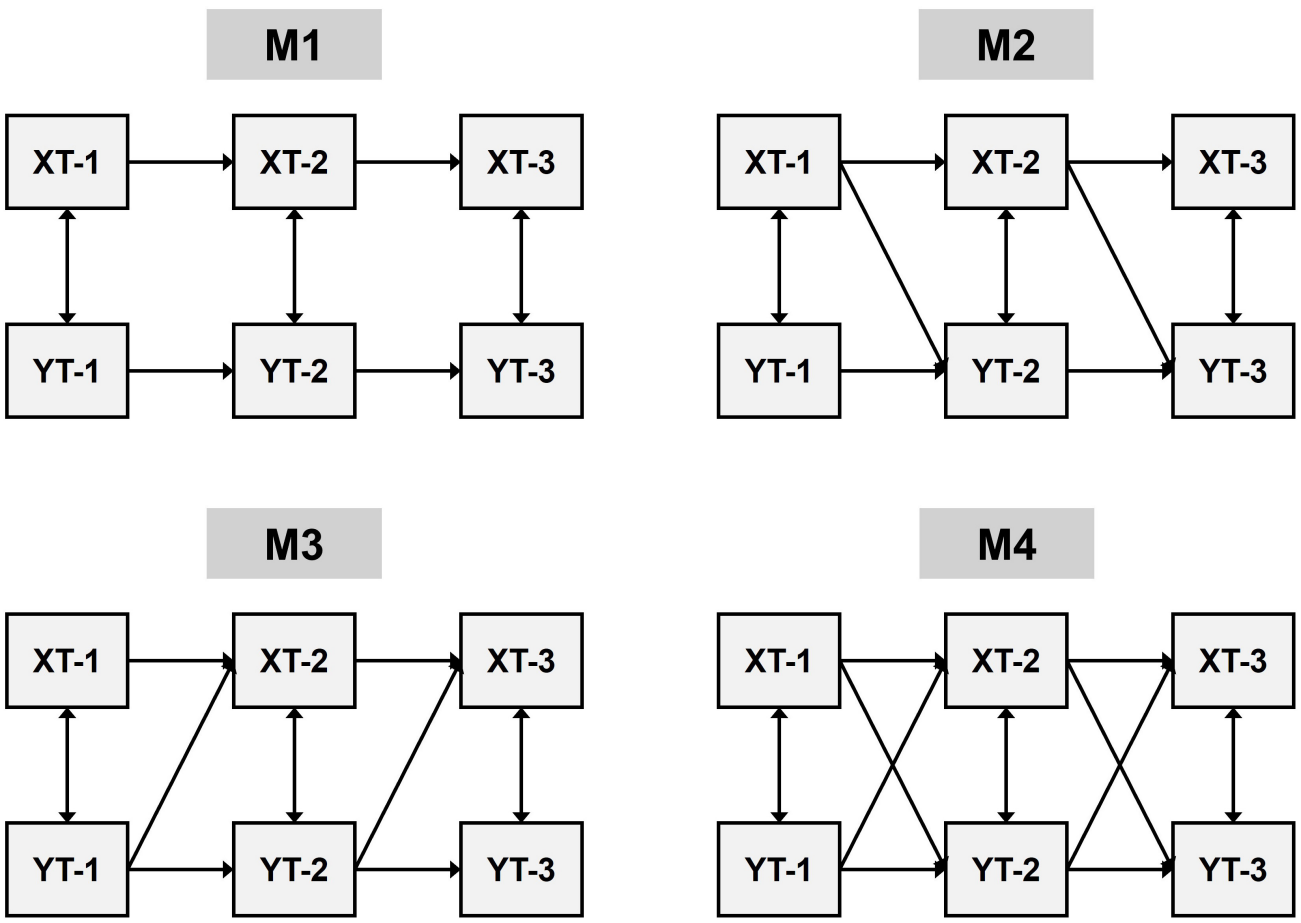
Cross-lagged panel regression model framework.

Using the cross-lagged effects test requires exploring the interrelationships between the independent variables and the dependent variable by building the following four models: (1) an autoregressive model M1 with only the independent and dependent variables. (2) a model M2 based on an autoregressive model with the independent variable X pointing to the dependent variable Y. (3) a model M3 based on an autoregressive model with the dependent variable Y pointing to the independent variable X. (4) a full model path M1 covering the M1, M2, M3 of the full model path M4. Detailed information is shown in Figure 4.

The parameters, path coefficients, degree of model fit, and model comparison of CLPM are shown in Table 4. The fit indices of all models except M3 are better than the autoregressive model of M1, and the chi-square differences between M2 and M4 and the autoregressive model M1 are significant (Δx^2^(2) = 98.02, *P* < 0.001, Δx^2^(4) = 371.10, *P* < 0.001). And the chi-square difference between M3 and autoregressive model M1 is not significant (Δx^2^(3) = 286.81, *P* > 0.05). This indicates that M2, M3, and M4 cross-regression models are all better than autoregressive model M1. Further analyses of M2 and M4 by competing models revealed that the chi-square difference between M2 and M4 was not significant (Δx^2^ = −269.41, *P* > 0.05). This indicates that there is no significant difference between M2 and M4 models. In the path coefficient analysis, it was found that SQ at T1 time period in the M4 full path model could not predict STE at T2 time period (β = −0.04, SE = 0.05, *P* = 0.358), and SQ at T2 time period predicted a smaller value of physical activity level at T3 time period (β = −0.06, SE = 0.07, *P* = 0.039). Based on judgement criteria such as the principle of model simplification and significant fit of path coefficients, model M2 can be considered the best model.

**Table 4:** Competition model fit index.

**Model**	***x* ^2^**	***df***	**CFI**	**GFI**	**SRMR**	**RMSEA**	**Δ*x*^2^**	**Δ*df***	***P***
M1	1,574.61	8	0.93	0.94	0.13	0.15			
M2	1,476.33	6	0.92	0.93	0.09	0.08	98.02	2	0
M3	1,286.27	6	0.91	0.95	0.08	0.14	286.81	2	0.059
M4	1,213	4	0.93	0.93	0.06	0.12	371.10	4	0

**Figure 5:**
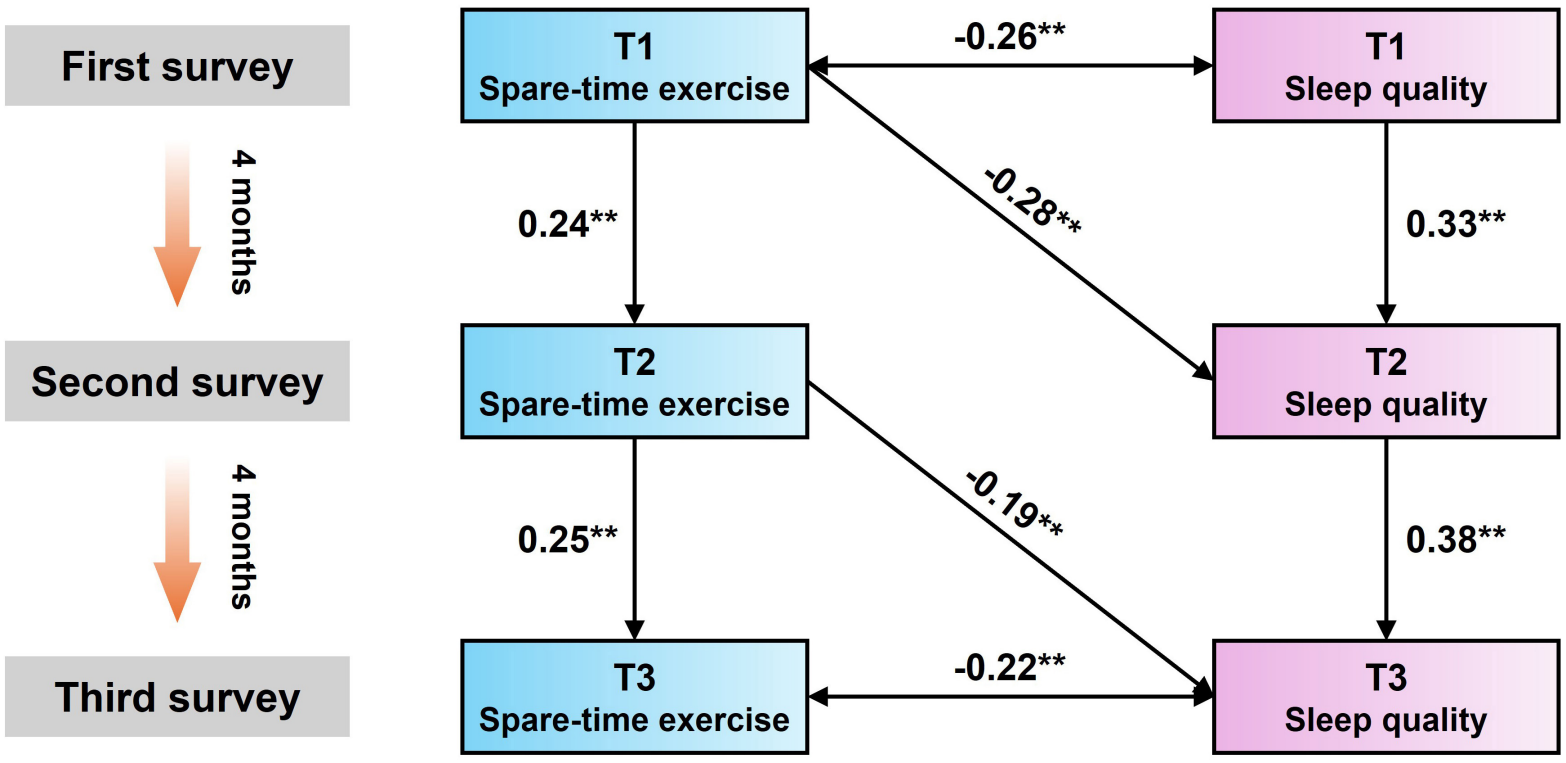
Cross-lagged panel model of spare-time exercise and sleep quality. * indicates *P* < 0.05, ** indicates *P* < 0.01.

The cross-lagged model of STE and SQ for secondary school students is shown in Figure 5. STE at the T1 time period significantly predicted SQ at the T2 time period (β = −0.28, SE = 0.05, *P* < 0.01). And STE in T2 time period also significantly predicted SQ in T3 time period (β = −0.19, SE = 0.05, *P* < 0.005). In summary, the results of both the latent variable growth model and the cross-lagged regression analyses indicated that STE was a significant negative predictor of SQ among secondary school students, and also begged to confirm the hypothesis that SQ does not predict physical activity level. These results are justified by the longitudinal trend trajectory and cross-lagged, and also re-emphasize that the hypothesis that STE affects secondary school students' SQ is stable and reliable.

## Discussion

In consideration of the significance and particularities pertaining to mental health, SQ, and emotion regulation within the secondary school student demographic, scholars have commenced a focus on their internal triggering mechanisms and developmental trajectories. Previous studies have predominantly concentrated on the impact of environmental factors, including the pressure from further education, interpersonal relationships during school, and familial issues. However, there is a paucity of research in this area, with only a small number of scholars having explored sports and recreation programmed in leisure life. In order to address this gap in the literature, this study adopts the latent variable growth model, parallel growth model and cross-lagged competition model to analyses the characteristics of the development trajectory of the variables and the causality argument in the longitudinal multiple time periods, and fully explores the internal generative mechanism. The study's findings indicate a tendency for STE levels among secondary school students to decrease over time, while SQ levels tend to rise. The intercept and slope of secondary school students' STE can predict the intercept and slope of SQ, respectively. Furthermore, the cross-lagged results suggest that STE levels of secondary school students can predict SQ levels in subsequent periods, and vice versa, although this relationship is not statistically valid.

The initial finding of this study was that secondary school students' STE levels exhibited a tendency to decline over time. In this study, STE levels gradually decreased with the number of follow-ups after the initial collection from T1 for all participants, thereby supporting hypothesis (I). This finding has been confirmed in previous studies. Seppänen found a strong positive correlation between STE and sleep quality by monitoring the participants' activity 24 h a day for two consecutive weeks. However, this effect started to level off after the 5th day (Seppänen et al., 2025). To further explore the temporal effects of STE and sleep, Marco then compared 86 RCT in a systematic review and found a U-shaped downward curve in the relationship between STE dose and sleep (Wang et al., 2025). This also highly overlaps with the results of the present study. The underlying reasons may be as follows: in real life, due to the short school system of secondary school students, each stage of junior high school and senior high school is only 3 years. This is particularly salient for third-year students, as graduation approaches and they face an increase in examinations and other significant events. These factors may contribute to a decline in secondary school students' emotional and psychological wellbeing, potentially leading to an escalation in negative emotions and maladjustment (Xu et al., 2025). Concurrently, the pressures associated with graduation and the deterioration of the living environment serve to exacerbate the anxiety experienced by secondary school students. In their quest for respite and transcendence, these students opt for sports and recreational activities as a means of alleviating their internalized anxieties (Galante et al., 2018). Nevertheless, the demanding curriculum of formal education, coupled with the challenges confronting the physical and mental wellbeing of graduates, engenders difficulties in the effective provision of treatment and attention. Consequently, the domains of STE and leisure activities are often overlooked, resulting in a diminution of secondary school students' enthusiasm for STE participation, which in turn leads to a decline in their perception of enjoyment and enthusiasm for physical exercise (Dishman et al., 2018). Moreover, the findings of this study indicated a negative correlation between the initial level of secondary school students' STE participation and the subsequent rate of decline. This suggests that groups with higher levels of secondary school students' STE participation exhibited a faster subsequent rate of decline. Conversely, groups of secondary school students with lower initial levels of STE demonstrated slower rates of subsequent decline. Consequently, this study hypothesizes that this phenomenon may be indicative of a “what goes around comes around” effect (Timo et al., 2016). Groups of secondary school students with high initial levels of STE are able to experience the physical and mental pleasure and personal fulfillment of fitness at the initial stage, and they are full of needs and expectations for the later stages of body perception (Nogg et al., 2021). However, as graduation approaches and distractions abound, it becomes challenging to maintain both physical and mental presence, consequently leading to a decline in the time and intensity of activities. This precipitous decline in STE levels is indicative of a marked contrast. The group of secondary school students who initially exhibited low STE levels were characterized by limited interpersonal relationships and neighborhood resources, minimal expectations regarding future PA values and demands, and a state of stable and continuous development. Consequently, the rate of growth in STE remained relatively unchanged in the face of subsequent deterioration and challenges from the external environment.

The second finding of this study was that the SQ of secondary school students showed a continuous increase over the three longitudinal data points. The reason for this phenomenon, according to the stress-sleep relationship theory, is that people under psychological stress are more likely to have perceptual distortions and trigger negative emotions, leading to a serious decline in sleep quality (Lo Martire et al., 2020). On the one hand, several studies have indicated that psychological stress is correlated with perceptual bias (McHugh et al., 2010). In the case of secondary school students, depression and anxiety may develop in an internalized spiral, along with sleep disturbances, until the negative emotions are effectively alleviated. For secondary school students in particular, secondary school is a semi-social developmental stage in which the student body is already aware of various levels of external challenges, which are also accompanied by symptoms such as mild sleep disturbances (Durlak et al., 2011). As students' progress through the educational system and encounter increasing academic demands, coupled with the development of complex emotional interrelations, the strategies and coping mechanisms they employ to manage external pressures may become increasingly ineffective. This may, in turn, result in a deterioration of sleep disorders in secondary school students over time (Wahlstrom and Owens, 2017). Conversely, while depression, anxiety and negative emotions have been demonstrated to precipitate sleep disorders in secondary school students, the extent to which these conditions influence others is contingent upon the temporal proximity of the onset of symptoms. In the nascent stage of the condition, the establishment of rapport and the cultivation of a warm environment have been shown to facilitate adaptation to the internal environment, with the objective of alleviating psychological discomfort. In this regard, it is imperative to facilitate the enhancement of interpersonal friendships in the early stage of the condition (Villanueva et al., 2024). However, in instances where middle school students encounter difficulties in coping with sleep disorders, they may resort to a series of adverse measures, which can further exacerbate their negative outlook and lead to depression. When confronted with the persistent impact of factors such as challenges in further education or emotional frustration, middle school students often receive limited support and positive feedback from their families and friends. This can lead to an intensification of self-regurgitation and a surge in negative emotions, thereby contributing to the gradual escalation of the sleep problem into a recurring pattern (Nicholson et al., 2021). The results of a longitudinal data survey further corroborated this perspective. Wang's longitudinal, long-term follow-up survey and analyses revealed a significant association between sleep disorders and depressive symptoms in secondary school students, with the severity of the association increasing in proportion to the level of depression (Wang et al., 2022). Theories and studies conducted by scholars have the potential to further clarify the characteristics and trends of SQ growth over time among secondary school students. Furthermore, this study found that the initial level (intercept) of secondary school students' SQ is significantly negatively correlated with the late growth rate (slope). This finding further underscores the notion that students with higher SQ levels exhibit a more gradual growth trajectory in the subsequent period, while those with lower initial levels demonstrate a faster rate of growth in the later stages. This phenomenon may be attributed to the capacity of secondary school students to self-regulate. The depression, anxiety and negative emotions exhibited by the group with higher SQ were in a symbiotic state, which had been gradually being moderated to cope with, and the individual's regulation and coping strategies were gradually in a peaceful state. Conversely, individuals with lower initial SQ levels exhibited a propensity to encounter difficulties in formulating positive coping strategies in response to sudden external stressors and experienced confusion regarding social barriers. This proclivity for negative emotions rendered them susceptible to heightened inner agitation and restlessness, consequently escalating the risk of SQ disorder development in subsequent stages (Lewis et al., 2018).

The third finding of this study was that the initial level of secondary school students' STE negatively predicted the initial level of secondary school students' SQ, i.e., groups with low levels of STE may be at higher risk for SQ. Therefore, hypothesis (II) can be accepted. The study hypothesis that this phenomenon may be due to the form of STE of most students, which are mostly group sports such as basketball or football. Numerous studies have demonstrated that group projects can effectively alleviate psychological distress and sleep disorders in secondary school students, surpassing the efficacy of closed-type skill projects. These studies also highlight the significant positive impact of group projects on the wellbeing of secondary school students (Gu et al., 2024). In the face of the deteriorating external environment and the continuous impact of the employment environment, moderate PA participation and group program diversion can help secondary school students to positively cope with anxiety, depression and sleep disorders in their lives, and to form correct cognitions and positive strategies. In this way, a more comprehensive and systematic mechanism of misperception reassessment and emotion regulation strategies can be established to help them cope with the discomfort caused by the decline of SQ (Glavin et al., 2022). Conversely, groups of secondary school students with low levels of physical activity participation have difficulty developing a positive and active outlook on life and resilience, and present higher levels of negative emotions in response to deterioration in the external environment (Nordberg et al., 2022). The concomitant rise in negative emotions and the gradual disintegration of interpersonal relationships result in a reemergence of distressing emotions in secondary school students, potentially precipitating outbursts of extreme emotions and maladaptive behaviors in the individual, thereby exacerbating the ongoing deterioration of sleep disorders and SQ (Fischer et al., 2020).

The fourth finding of this study was that the initial level of STE among secondary school students may also have an impact on the rate of SQ development. The study demonstrated that the lower the level of secondary school students' participation in STE, the faster their rate of SQ growth; conversely, the higher the initial level of physical activity group, the lower the risk of SQ. This phenomenon can be explained by Bandura's theory of self-regulation, which suggests that an individual's subjective psyche renders their behavior vivid and selective, thereby influencing their cognition, emotion and behavior (Bandura, 2001). The present study posits that the participation of STE provides insights and pathways to elucidate the internal influence mechanism of sleep disorders. Individuals must continuously enhance their willpower, mental intelligence, and emotional regulation in the process of social adaptation enhancement. The involvement in sports and the sense of identity gained in group programs furnish opportunities and fundamental conditions for the enhancement of their psychological fitness (Smith and Merwin, 2021). The absence of STE and recreational programs in secondary schools has been demonstrated to be a contributing factor to the accumulation of internal anxiety and depression among students. In order to alleviate the internal discomfort and emotional agitation experienced by the individual psyche, it is recommended that secondary school students engage in experiential projects during their leisure time. This approach aims to facilitate the correct cognition of erroneous emotional expressions and the improvement of regulatory strategies (Fu et al., 2023). In the initial stages, groups exhibiting a low level of STE involvement demonstrate a limited capacity to comprehend and adapt to external perceptions of misperceived emotions. This phenomenon precipitates the rapid escalation of negative and hostile emotions, thereby augmenting the likelihood of the utilization of immature defense mechanisms. The selection of such defense strategies accelerates the prevalence of denial behaviors, such as avoidance and concealment, resulting in increased selective withdrawal and emotional rumination (Walsh et al., 2020). The operation of this mechanism of selective withdrawal has been demonstrated to result in individuals demonstrating a greater propensity to evade the issue and instead resort to negative behaviors, such as self-absorption or avoidance (Sege et al., 2023). As the psychological systems of secondary school students become imbalanced and their external supportive environment shrinks, their ability to regulate their emotions gradually declines. This decline is more likely to cause depression, anxiety and other negative emotions, and to accelerate the exposure of SQ problems. Conversely, individuals with a high level of participation in STE interpersonal interactions are more likely to receive recognition, praise and assistance from others. The increase in social support and emotional communication helps individuals to obtain appropriate emotion regulation strategies and coping mechanisms, which can effectively alleviate the psychological discomfort and external pressures encountered in daily life (Dedoncker et al., 2021; Scrivano et al., 2023). From an individual development perspective, such regulatory strategies and coping mechanisms can effectively assist individuals in adapting to social development and overcoming sleep disorders, thereby ensuring the physical and mental wellbeing of students.

Moreover, the study concluded with the finding that the rate of decline in STE predicted SQ growth among middle school students across the three time periods. This suggests that the faster the decline in STE participation levels among the group of middle school students, the faster their SQ grew. Conversely, a slower decline in STE levels was associated with slower SQ growth. These results are consistent with the findings of previous studies and with the Stress-Sleep Relationship Theory (Zhang et al., 2024). This study posits that the sustained and smooth participation of secondary school students in STE can effectively enhance their psychological and social adaptability, thereby facilitating the development of a more extensive network of interpersonal relationships and social recognition. Consequently, it improves the individual's capacity to regulate emotions and employs regulatory strategies to cope with external stress, thus alleviating internal turmoil and problems in SQ. Conversely, the rapid decline in STE participation has been shown to result in a loss of opportunities for communication, collaboration, and support, leading to social disorders and agitated behaviors. This, in turn, has been demonstrated to accelerate the loss of self-restraint and efficacy, and to trigger negative emotions, which ultimately leads to a rapid increase in SQ. The present study investigated the causal relationship between STE and SQ of secondary school students through regression effect analysis following cross-tabulation. The findings demonstrated that secondary school students' STE exhibited a significant negative prediction of SQ in the subsequent period, and vice versa, with non-significant and low effect values. Consequently, hypothesis (III) can also be accepted. These results further expand the interaction mechanism between STE and secondary school students' sleep and support the judgement that STE can predict SQ in the previous hypothesis. The findings are consistent with the results of scholars who have shown that high levels of participation in sports programs can positively enhance the sense of individual efficacy, obtain more social support and psychological assistance, and help to form a benign emotional regulation strategy, thereby dissolving the sleep disorder problem caused by emotional discomfort (Strahler et al., 2020; Vella et al., 2018). Conversely, reduced engagement in sports and leisure activities during secondary school has been demonstrated to impede students' subjective initiative, emotional adjustment, and social support. This, in turn, has been shown to prompt individuals to adopt a withdrawn and ruminative response to external pressures and psychological distress, thereby exacerbating the rapid development of SQ (Schuch et al., 2019; Difrancesco et al., 2019).

To summarize, while there is evidence that PA improves SQ, the reality is that secondary school students engage in STE more frequently. The findings of this study indicate that the initial high level of STE has the most significant improvement effect on SQ. However, it should be noted that the effect of STE on SQ is diminished by the fact that many exercise enthusiasts' STE levels decrease over time. The core of STE's effect on SQ is therefore its “sustained and quantitative” nature. The most effective intervention for SQ is consistent and regular use of STE at a certain intensity. It is therefore necessary to avoid exercising fatigue when choosing sports and daily intensity. This is due to the fact that exercise fatigue can lead to the accumulation of lactic acid and intracranial free radicals. Furthermore, it can result in a decrease in exercise adherence and loss of interest in exercise (Chausse et al., 2024). In severe cases, this may also lead to the occurrence of sports injury or rhabdomyolysis (Sabouri et al., 2024). Consequently, in accordance with the principles of appropriate load and motivation in sport (Impellizzeri et al., 2022; Nielsen et al., 2024), this study proposes that the rating of perceived exertion (RPE) or the feeling scale (FS) can serve as effective tools for the quantitative assessment of exercise condition prior to exertion (Mortimer et al., 2024; Prydz et al., 2024). The intervention also aims to increase the intensity of exercise in a single session or the duration of PA per week, where possible, in subsequent STEs. This intervention is conducive to the sustained and effective improvement of SQ and mental health in secondary school students.

## Limitations and suggestions for future research

There are still some shortcomings and areas to be improved and remedied in this study. Firstly, the method of subjects for the variables used in this study is the questionnaire filling method. Although the anonymity of the questionnaire entries and the objectivity and fairness of the subjects were strictly controlled in the planning of the subjects in the early stage of the study, the subjectivity of the individual in filling in the questionnaires and the bias in the understanding of the knowledge cannot be guaranteed, which inevitably resulted in systematic bias due to the formation of individual differences. In the follow-up study, we can use physiological instruments and neurological instruments to obtain more objective and meaningful experimental data.

Firstly, although this study discusses the effects of STE on the sleep of middle school students, the samples were taken from six middle schools in Beijing and may not be fully representative of middle school students due to regional, ethnic or customary differences. The team will investigate more schools and populations in more areas in future studies to address this issue. Secondly, confounding variables such as academic stress and family support were already present in this study. Therefore, the present study addressed this issue through this longitudinal follow-up study. Regarding the limitations of using self-report rather than objective measures, the two most internationally recognized scales were used for inclusion in this study. This was done in order to maximize the reliability and validity of the questionnaire. As far as the division into different physical activities is concerned, since the core of this study was to first evaluate the temporal effects and characteristics of STE on sleep in secondary school students. Therefore, no between-group control study was conducted, and the realization of STE as a whole. A CLPM based on two variables was established. However, we will continue to investigate the differences between types of sport in future studies using cross-sectional surveys with large samples or intervention experiments.

Second, the sample size is an important indicator of longitudinal research analysis and authority, in the process of the subjects to ensure that the attrition rate is reduced and fill in the quality of the next round of the way to carry out the interval of 4 months, but only three times to do the subject research, the follow-up did not carry out continuous tracking research, cannot be ruled out the subsequent 4th or 5th variable development trend and trajectory characteristics of the change may be; In addition, the existing In addition, the existing literature is mostly baseline and short-term data observations, all of which are linear development trends. The three observations can only be fitted to a latent variable growth model, which is no substitute for longitudinal results and trajectory characteristics obtained from tracking studies over multiple periods.

Therefore, in future studies, it is necessary to analyze the tracking data over a number of consecutive periods to sort out the development trends and mechanisms of the variables, in order to obtain a more authoritative and precise mechanism of the development of STE and SQ among secondary school students.

## Conclusion

(1) Initial levels of STE in secondary school students negatively predicted the rate of initial and subsequent development of SQ, and high levels of STE may be a protective factor in the development of SQ levels. (2) The effect of STE on SQ is more stable and negatively predicts SQ for the next time in all three measurements. Conversely, SQ has no significant effect on changes in STE. (3) STE decreases over time, so continued exercise and intensity are key to improving SQ.

## Data Availability

The original contributions presented in the study are included in the article/supplementary material, further inquiries can be directed to the corresponding author.
